# Effect of Phosphoric Acid on the Properties of Sodium Bentonite and Its Mechanism

**DOI:** 10.3390/molecules30040843

**Published:** 2025-02-12

**Authors:** Jiandi Liu, Yanzhi Meng, Yuze Zhang, Xiangyu Ji, Zhenhua Zheng, Luyan Wang, Wenjuan Guo, Meishan Pei

**Affiliations:** School of Chemistry and Chemical Engineering, University of Jinan, Jinan 250022, China; 17860282296@163.com (J.L.); 19906445534@163.com (Y.M.); 13386410911@163.com (Y.Z.); 19819790936@163.com (X.J.); luv179366@163.com (Z.Z.)

**Keywords:** H_3_PO_4_, sodium bentonite, unconfined compression strength, zeta potential

## Abstract

Expansive soils, widely distributed in nature, often pose challenges to construction stability due to their low unconfined compressive strength (UCS), poor shear strength, and high expansibility. This study investigates the application of phosphoric acid (H_3_PO_4_) in modifying sodium bentonite, focusing on its effects on the mechanical properties and swelling behavior of bentonite, as well as the underlying mechanisms. H_3_PO_4_ was added to bentonite at mass ratios of 1% to 8%. Compared to unmodified bentonite, the plastic index of the modified bentonite decreased by 39.9%, and the UCS value increased by 92.24% when the H_3_PO_4_ dosage was 2%. Notably, at an H_3_PO_4_ dosage of 8%, the free swelling rate of the modified bentonite decreased by 38.1% relative to the control sample, and the cohesion increased by 165.35%, indicating significant improvements in both the expansibility and bearing capacity of modified bentonite. The results on the physical and chemical properties of modified bentonite revealed an ion exchange involving hydrogen ions from H_3_PO_4_ and metal cations in sodium bentonite. The zeta potential of bentonite decreased with H_3_PO_4_ addition, reflecting a reduction in the double electric layer thickness due to hydrogen ion exchange with metal cations. This enhanced the gravitational attraction between soil particles, leading to their closer proximity and a significant increase in the UCS value of the modified soil. Additionally, the XRD results confirmed that the addition of H_3_PO_4_ facilitated the formation of a new mineral, aluminum phosphate, which is hard and insoluble, filling soil pores, contributing to its densification. This study demonstrates that H_3_PO_4_ can effectively enhance the swelling resistance and strength of sodium bentonite, offering a promising method to improve its application performance.

## 1. Introduction

Expansive soil is widely distributed across the globe, particularly in China, where it extensively covers regions from the northeast to the southwest. When utilized as foundation or subgrade material, expansive soil frequently exhibits uneven expansion deformation, displacement, or cracking due to its inherently low unconfined compressive strength, low shear strength, and pronounced expansiveness [1]. These characteristics can result in subsidence and landslides in subgrades, as well as the rupture and deformation of underground pipelines. Among expansive soils, sodium bentonite is particularly problematic due to its strong hydrophilicity and significant swelling potential [2]. These properties, while beneficial in some applications, pose severe challenges in construction and geotechnical engineering, as they lead to poor mechanical stability and long-term durability issues. Therefore, it is essential to develop effective modification techniques to improve the engineering properties of expansive soils, particularly sodium bentonite, to meet stringent construction requirements.

When expansive soils are encountered at a construction site, two principal treatment methods are generally considered: complete replacement of the expansive soil deposits or remediation via soil stabilization techniques. Considering the significant expenses associated with the replacement method, researchers have increasingly shifted their focus towards soil remediation utilizing admixtures. Commonly utilized soil amendments include cement [3] lime [4,5], fly ash [6], various industrial by-products [7], and sulphonated oils [8]. Tamer et al. [3] examined the relative contributions of lime cementation and stress absorption to the swelling potential of two lime-treated expansive soils under various curing conditions. Nalbantoğlu [4] explored the effects of fly ash treatment on soil texture and plasticity, specifically through the reduction in clay particle dosage, leading to decreased plasticity index and swelling potential. Ghobadi [5] conducted geotechnical and mineralogical studies on lime-treated clays, focusing on the impact of pH changes on shear strength parameters. Almuaythir et al. [9] investigated the impact of various industrial by-products, including silica fume, cement kiln dust, calcium carbide residue, rice husk ash, and ground granulated blast furnace slag, on the physical, mechanical, and microstructural properties of soils under different curing conditions. A commercially available sulphonated oil was used by Soltani et al. [10] to treat highly expansive soils, aiming to enhance their swelling characteristics and strength properties for stabilization purposes.

Although these soil curing agents have demonstrated effectiveness as stabilizing materials, they exhibit certain limitations. For example, the stabilization of expansive soils using lime or cement mixes not only incurs high costs but also poses significant environmental risks. Utilizing industrial waste for soil stabilization is a promising method that enhances the engineering properties of expansive soils while contributing to waste management and environmental protection. Nevertheless, this approach is labor-intensive and time-consuming, thereby increasing construction costs. Moreover, while stabilization with sulphonated oils improves swelling properties, its limited mixing volume hinders construction efficiency, and the prohibitively high cost further exacerbates economic concerns. Therefore, there is an urgent need to develop new expansive soil curing agents that are both environmentally friendly and capable of effectively mitigating the expansion properties of expansive soils.

H_3_PO_4_ has been reported by researchers as a soil curing agent. Medina et al. [11] studied the effect of H_3_PO_4_ on lateritic soils, with kaolinite as the main component, for road and airport construction in the tropics. Eisazadeh et al. [12] investigated H_3_PO_4_ stabilization of sandy clays, with kaolinite as the main component, to determine the unconfined compressive strength for different curing times and immersion times. Ingles [11] used either H_3_PO_4_ or phosphate as a chemical stabilizer only for acidic soils. He et al. [13] explored the mechanical properties and microstructure of geopolymer (PAFG) samples prepared at different low H_3_PO_4_ concentrations, as well as the environmental and economic impacts. Ede [14] studied the use of H_3_PO_4_ for stabilizing black soils with the addition of micro steel fibers to improve the California bearing ratio (CBR) value.

However, the effects of H_3_PO_4_ on the stabilization of expansive soils have not been investigated independently. Moreover, previous studies on H_3_PO_4_ primarily focused on acidic soils predominantly composed of kaolinite. In contrast, sodium bentonite, which is alkaline, consists mainly of montmorillonite with minor amounts of kaolinite and illite. According to the technical specification for highway roadbed construction, classification of JTG/T3610-2019, sodium bentonite belongs to strong expansion soil. Consequently, in this work, sodium bentonite was selected to study the stabilization mechanism of H_3_PO_4_ in soil. This investigation aims to deepen our understanding of the internal mechanisms governing H_3_PO_4_-induced soil stabilization, thereby exploring the feasibility of utilizing phosphoric acid as a curing agent for expansive soils. Additionally, it provides valuable insights and an effective reference for addressing engineering challenges associated with expansive soil.

## 2. Results and Discussion

### 2.1. Analysis of Limit Water Content Test

The liquid limit, plastic limit, and other water content parameters are crucial physical properties of soil that not only reflect its water-holding capacity and strength but also characterize the extent to which water affects the engineering properties of soil.

The experimental results are presented in Table 1, where the addition of H_3_PO_4_ results in a decrease in liquid limit (LL), an increase in plastic limit (PL), and ultimately a decrease in plasticity index (PI). When H_3_PO_4_ dosage is 2%, the PI value of modified bentonite decreases to 52.9%, representing a reduction of 39.9% compared to the unamended soil without phosphoric acid. Several mechanisms contributed to the reduction in soil PI, including ion exchange reactions, flocculation, and agglomeration of soil particles. The first two processes occur rapidly and have an immediate impact on plasticity [15]. These mechanisms elevate the electrolyte concentration and cation valence within the treated soil, thereby diminishing repulsive forces between soil particles. Consequently, this causes the contraction of soil skeleton, promotes the aggregation of soil particles, and reduces the water holding capacity of soil [6], resulting in a decline in PI of soil. This reduction not only signifies an enhancement in the shear strength of the modified soil, thereby improving its resistance to shear damage under external forces, but also leads to decreased compressibility. The improved compressibility enhances the soil’s bearing capacity and reduces the risk of foundation settlement. Additionally, this modification results in better workability and improved operability of the modified soil. It is crucial to emphasize that the relationship between hydrophilicity and PI of expansive soil demonstrates that an increase in PI correlates with a decrease in hydrophilicity, thereby diminishing the expansibility of the soil. As a result, the incorporation of H_3_PO_4_ markedly suppressed the expansion of bentonite.

### 2.2. pH and Electrical Conductivity

The pH and EC values of the solution determine the mode of dissociation of molecules on the surface of the particle, which, in turn, determines the sign and number of charges on the surface of the particle. The latter also affects the thickness of the diffusion layer.

**Figure 1:**
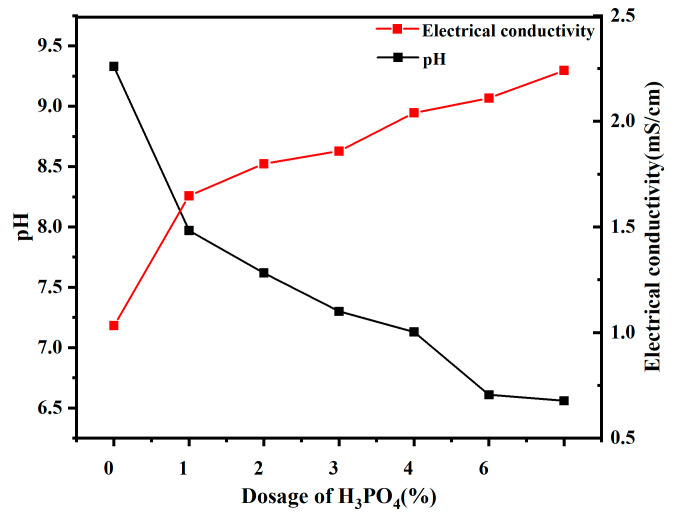
Effect different dosages of H_3_PO_4_ on physico-chemical properties of bentonite.

The changes in soil pH and electrical conductivity at different H_3_PO_4_ dosages are illustrated in Figure 1. As the dosage of H_3_PO_4_ gradually increased, there is a gradual decrease in soil pH from 9.34 to 6.56. This decline can be attributed to the reaction between H_3_PO_4_ and substances such as aluminum trioxide present in the soil, resulting in a reduction in soil pH. Additionally, it is noteworthy that soil pH plays a pivotal role in determining the thickness of the double layer on the surface of soil particles [16]. Simultaneously, an increase in electrical conductivity of the soil is observed from 1.033 mS/cm to 2.24 mS/cm due to elevated dosages of H_3_PO_4_ and soluble ions, leading to enhanced conductivity values. This rise in conductivity creates favorable conditions for reducing zeta potential, diminishing repulsive forces, and promoting the formation of aggregation structure [15].

### 2.3. Analysis of Cation Exchange Capacity (CEC)

Bentonite is a clay mineral predominantly composed of montmorillonite. Montmorillonite exhibits a 2:1 “sandwich” layer structure, characterized by an aluminum–oxygen octahedron intercalated between two silica tetrahedra. These layered structures stack to form the crystal lattice and contain exchangeable cations such as potassium, sodium, calcium, and magnesium either between the layers or on the surface. The essential characteristics of bentonite are fundamentally defined by the chemical composition, crystal lattice structure, and mineralogical composition of montmorillonite. Due to its unique structural properties, bentonite demonstrates exceptional expansibility and cation exchange capacity (CEC), which is an important parameter in characterizing the expansion of soils.

In this study, 2% H_3_PO_4_-modified sodium bentonite was selected for investigation due to its optimal performance in terms of compressive strength, shear strength, and the lowest plasticity index (PI), as well as a significant reduction in free swell ratio. The results indicate that the CEC value of the modified bentonite with 2% H_3_PO_4_ was 0.77 mmol/g, representing a 9.4% decrease compared to that of the unmodified control soil (0.85 mmol/g). During the testing process, methylene blue forms monovalent organic cations in aqueous solution, which have the ability to displace exchangeable cations in soil minerals such as bentonite. Upon the addition of H_3_PO_4_, a significant influx of hydrogen ions into the bentonite leads to the initial exchange of adsorbed cations within the soil. Consequently, this reduces the number of methylene blue ions exchanged, thereby demonstrating a decrease in the CEC of the modified bentonite.

It is important to note that soil expansion is closely associated with the hydration of interlayer cations, where stronger hydration results in more pronounced clay expansion. The hydration of soil particle surfaces is primarily driven by the interaction between water molecules and the negatively charged soil colloidal surface, as well as the cations adsorbed around it. As the number of cations decreases, their attraction to polar water molecules weakens, leading to a reduction in the amount of water molecules adsorbed on the soil particle surface, thus decreasing the hydration energy of soil particles [17]. The observed reduction in CEC in modified bentonite signifies a decline in the number of exchangeable cations, which, in turn, diminishes clay swelling caused by interlayer cationic hydration. This finding aligns with the observed decrease in PI.

### 2.4. Analysis of Zeta Potential Test

The zeta potential is mainly related to the pH and electrolyte concentration of the soil particles [18]. Changes in pH and electrolyte concentration on the surface of the grains lead to changes in the zeta potential of the sodium bentonite surface. The zeta potential is closely related to the Coulomb repulsion between the sodium bentonite particles: the larger the absolute value of zeta potential, the stronger the electrostatic repulsion between the particles; conversely, the smaller the absolute value of zeta potential, the weaker the electrostatic repulsion between the particles [19].

**Figure 2:**
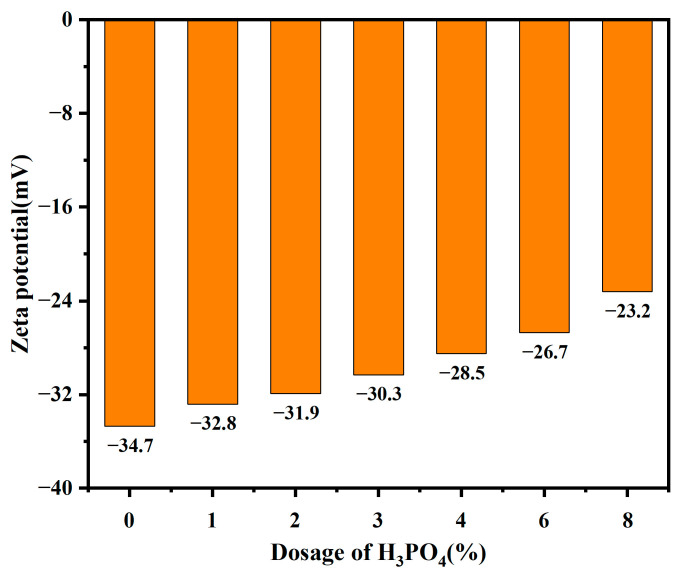
Effect of H_3_PO_4_ dosages on zeta potential of bentonite.

The zeta potential of modified bentonite with varying dosages of H_3_PO_4_, as depicted in Figure 2, reveals a negative surface charge on the surface of both soil particles in sodium bentonite and modified samples. Although a small amount of positive charge may be present at the fracture of sodium bentonite crystals, its magnitude is much lower than the surface-dominated negative charge. As the H_3_PO_4_ content increases, the concentration of H⁺ in solutions rises, decreasing the absolute value of the zeta potential through the following mechanisms: (1) cation exchange: H⁺ replaces Na^+^ or other ions in the diffusion layer, decreasing the Stern layer potential, and thus the zeta potential at the shear surface. (2) bilayer compression: H⁺ increases the ionic strength, shortening the Debye length and compressing the diffusion layer [20]. In addition, adsorption is also an important factor affecting the double electric layer. Specifically, the adsorption of H_3_PO_4_ onto the surface of bentonite modifies the thickness of the diffuse layer. This occurs because the negatively charged ions on the bentonite surface attract and adsorb phosphate through electrostatic forces, partially obstructing some of the pores within the bentonite layers. Consequently, this leads to a reduction in both the specific surface area and porosity, neutralizing the negative charges on the clay surface and thereby decreasing the absolute value of the zeta potential [21]. These processes act synergistically to weaken the inter-particle electrostatic repulsive force while enhancing the van der Waals attraction due to reduced spacing. Consequently, the aggregation and close packing of particles are promoted, leading to the formation of denser and more bonded agglomerates, which increase the mechanical strength of the soil [22].

### 2.5. Analysis of Free Swelling Test

The free swelling rate of soil serves as a direct indicator of its expansion potential. As shown in Table 2, the free swelling rate of modified bentonite is significantly lower than that of unmodified bentonite. With increasing H_3_PO_4_ dosage, the free swelling rate of the modified bentonite decreases progressively. Specifically, compared to the unmodified bentonite, the free swelling rates for modified bentonite with 2% and 4% H_3_PO_4_ decreased by 23.8% and 28.6%, respectively. This reduction indicates the inhibitory effect of H_3_PO_4_ on the soil’s swelling capacity. Soil expansion is primarily governed by two mechanisms: the lattice expansion of soil minerals and the double electric layer theory. In this study, we focus on the double electric layer theory. According to Figure 2, the absolute value of the zeta potential of soil particles decreases with increasing H_3_PO_4_ dosage, indicating a reduction in the thickness of the diffuse layer within the double electric layer. Consequently, this leads to a decrease in weakly bound water content. The reduced weakly bound water content correlates with diminished soil expansion capacity, thereby resulting in a lower free swelling rate in the modified bentonite.

### 2.6. Unconfined Compressive Strength Test

The unconfined compressive strength (UCS) of soil is a critical parameter that directly reflects its intrinsic mechanical properties and thus serves as a standard metric for evaluating soil strength.

**Figure 3:**
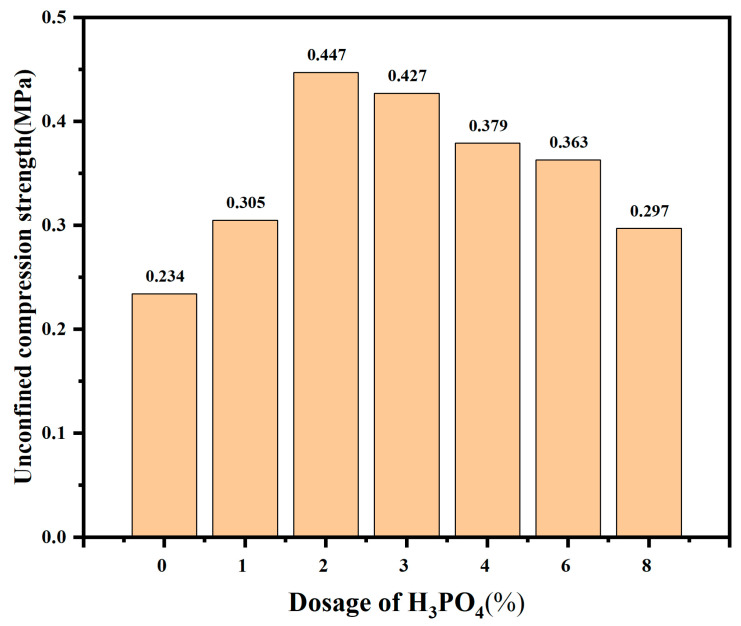
Unconfined compression strengths of bentonite with different dosages of H_3_PO_4_.

The results of the UCS test for soils with varying H_3_PO_4_ dosages are depicted in Figure 3. Upon reaching a H_3_PO_4_ dosage of 1%, the UCS of the improved soil exhibits a marginal increase compared to the untreated soil, rising from 0.234 MPa to 0.305 MPa. Subsequently, as the H_3_PO_4_ dosage ranged between 1% and 2%, there is a significant enhancement in UCS for the improved soil, escalating from 0.305 MPa to 0.447 MPa. The experimental findings demonstrate that adding 2% H_3_PO_4_ to the sodium bentonite results in a significant enhancement of its UCS by approximately 92.24% compared to untreated soil. Conversely, when the H_3_PO_4_ dosage exceeded 2%, a decreasing trend in UCS is observed for the modified bentonite, declining from 0.447 MPa to 0.297 MPa. Notably, the UCS values of all modified bentonite are significantly higher than that of the untreated soil, attributed to the formation of gelled aluminum phosphate, which exhibits enhanced strength. At a higher H_3_PO_4_ dosage, the UCS of the modified bentonite decreases, possibly due to the reaction of H_3_PO_4_ with calcite in sodium bentonite.

### 2.7. Direct Shear Experiment

The stability of soil slopes, retaining walls, and deep foundation walls, as well as the bearing capacity of foundations, are all closely related to shear strength, making it necessary to carry out direct shear tests.

**Figure 4:**
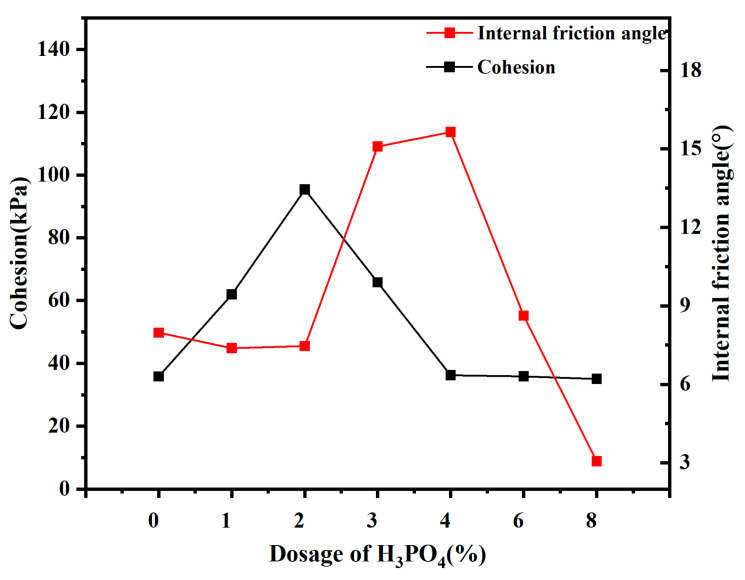
Shear strength parameters of bentonite with different dosages of H_3_PO_4_.

The results of the direct shear experiment on soil with varying H_3_PO_4_ dosages are presented in Figure 4. With increasing H_3_PO_4_ dosages, the cohesion of sodium bentonite initially increased and then decreased. In the range of H_3_PO_4_ dosage from 0 to 2%, the cohesion steadily went up at a certain growth rate, with sodium bentonite exhibiting an increase in cohesion from 35.82 kPa to 95.38 kPa. However, further increase in H_3_PO_4_ dosage leads to a decrease in cohesion from 95.38 kPa to 65.1 kPa. The increase in soil cohesion indicates that the addition of H_3_PO_4_ enhances soil cohesion through a series of physicochemical reactions with soil particles.

As shown in Figure 4, compared with untreated soil, the absolute value of the zeta potential of modified bentonite decreases. This reduction suggests a lower particle surface charge, a thinner double electric layer, and a reduced thickness of the bound water film. As a result, the reduced distance between soil particles leads to weakened repulsive forces and strengthened attractive forces, promoting particle aggregation and proximity. Consequently, the soil body becomes denser as the bonding force between soil particles strengthens, which alters their contact patterns [23,24]. The highest cohesion is observed when H_3_PO_4_ dosage reaches 2%, indicating the optimal interconnection among soil particles. However, the cohesive force decreases as the H_3_PO_4_ concentration increased from 2% to 8%, attributed to the disruption of tight particle contact caused by excessive acid [25]. To some extent, the increase in the internal friction angle is due to the decrease in the thickness of the bilayer and the closer bonding between the particles. The friction increases when relative sliding occurs between soil particles, thus increasing the internal friction angle. In cohesive soils with finer textures, lamellar soil mineral particles are typically aligned face to face due to their small size. Under horizontal shear forces, these particles predominantly undergo face-to-face sliding motion, resulting in minimal and negligible increments in internal friction angle.

### 2.8. Fourier-Transform Infrared Spectra

The positions and intensities of the absorption peaks in Fourier-transform infrared (FTIR) spectra serve as indicators of the molecular structure’s characteristics, thereby enabling the identification of the structural composition of unknown substances or the determination of their chemical groups.

**Figure 5:**
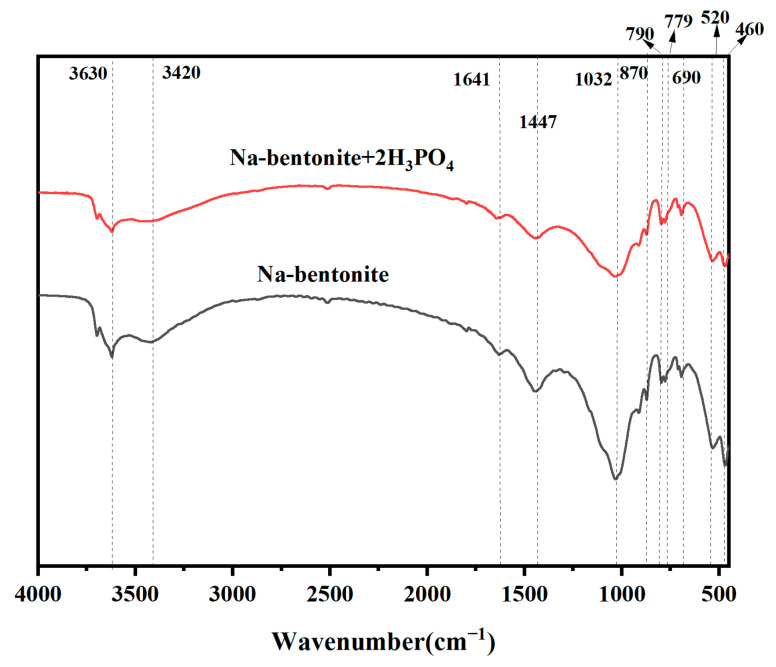
FIIR spectra of bentonite and modified bentonite with 2% H_3_PO_4_.

The FTIR results of sodium bentonite with a 2% H_3_PO_4_ dosage and the blank sodium bentonite are shown in Figure 5. The FTIR spectra of three samples exhibit similar shapes, with slight variations in peak intensities, suggesting that the addition of H_3_PO_4_ has influenced the functional groups present in the soil to some extent. Specifically, at 3630 cm^−1^, there is a stretching vibration corresponding to structural -OH in sodium bentonite, while the peak at 3420 cm^−1^ represents hydrogen bond binding in the hydroxyl group [26]. Moreover, the peaks at 1641 cm^−1^ represent the bending vibration of H-O-H in water; at 1447 cm^−1^, the telescoping vibration of O-C-O attributes mainly to the presence of carbonates, and bands at 871 cm^−1^ and 713 cm^−1^ correspond to characteristic peaks of calcite. Peaks observed at both 790 cm^−1^ and 779 cm^−1^ represent telescoping vibrations related to Si-O bonds in quartz. Additionally, a peak at 690 cm^−1^ indicates the bending and deformation of Si-O bonds. Furthermore, absorption peaks detected at 1032 cm^−1^ suggest stretching vibrations associated with Si-O-Si(Al) and Al-O-P bonds, which mean the formation of aluminum phosphate [27,28], whereas those found at both 520 cm^−1^ and 460 cm^−1^ correspond to bending vibrations related to Si-O-Al and Si-O-Si [29]. Compared to the untreated bentonite, the addition of H_3_PO_4_ weakens the peaks at 3400 cm^−1^ versus 1640 cm^−1^, indicating that the amount of excessively weakly bound water adsorbed in the interlayer became less. Thus, it further proves that the hydrating water film between the modified bentonite layers becomes significantly thinner.

### 2.9. X-Ray Diffraction Patterns

**Figure 6:**
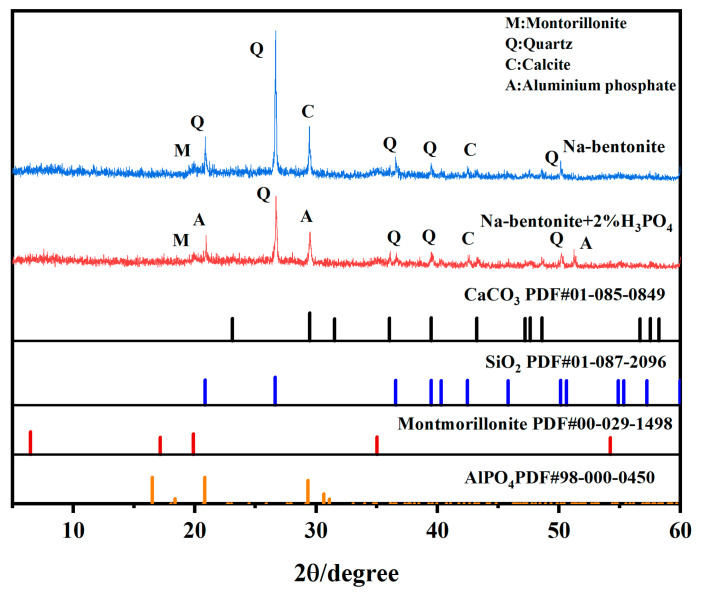
XRD patterns of bentonite and modified bentonite with 2% H_3_PO_4_.

X-ray diffraction pattern (XRD) is a sensitive, adaptable, and reliable technique for providing qualitative information on the crystalline compounds in soils [30,31]. XRD results of bentonite and H_3_PO_4_-modified bentonite at 2% H_3_PO_4_ dosage are shown in Figure 6. It reveals that the addition of H_3_PO_4_ led to a modification in the peak intensities and the emergence of a new diffraction peak at 2θ = 21.1°, 29.5°, and 51.2°, signifying the presence of newly formed mineral aluminum phosphate in modified bentonite [14]. The aluminum phosphate produced is hard and insoluble in water, thus increasing the compressive and shear strength of the soil. Additionally, calculating the interplanar distances of characteristic peaks using Bragg’s formula, it can be found that in the crystal structure of montmorillonite, the interplanar spacing derived from the diffraction peak at 2θ = 19.89° for modified bentonite is 4.45 Å, which is marginally smaller than the 4.46 Å observed for unmodified bentonite. This slight reduction is attributed to the addition of H_3_PO_4_, which thins the hydration layer between the bentonite layers, thereby decreasing the interlayer spacing. However, the extent of this reduction is minimal, indicating that the addition of phosphoric acid largely preserves the original crystal structure of the soil.

### 2.10. Micromorphology

**Figure 7:**
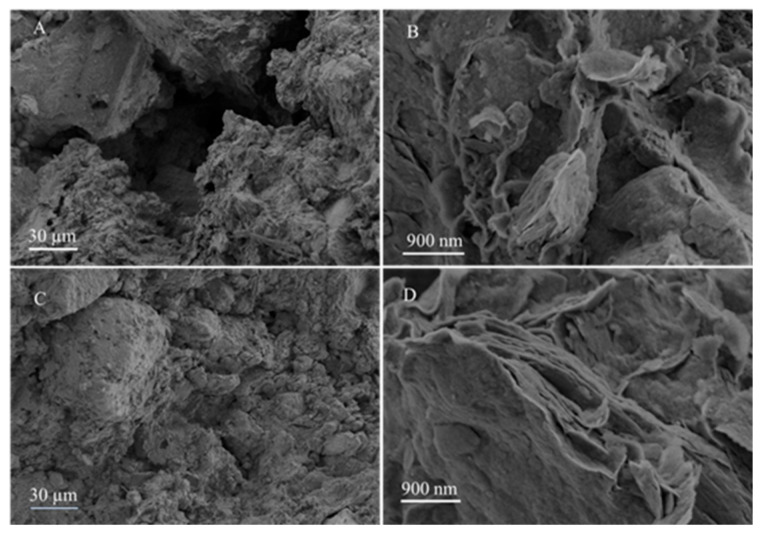
SEM images of bentonite (**A**,**B**) and modified bentonite with 2% H_3_PO_4_ (**C**,**D**) after cured for 7 days.

The scanning electron microscopy (SEM) test facilitates the acquisition of detailed microscopic information, such as surface morphology, micro-pore structure distribution, and particle size variation, of samples under high magnification conditions. The SEM images in Figure 7 present the micromorphologies of bentonite and the modified bentonite with 2% H_3_PO_4_ dosage. The untreated sodium bentonite exhibits a higher number of gaps compared to the modified bentonite, with the latter displaying deeper fissures. Upon the addition of H_3_PO_4_, a significant reduction in gap size and interparticle distance is observed. This phenomenon can be attributed to the decrease in zeta potential, which leads to a thinner bilayer, a reduced thickness of the bound water film, and consequently, tighter particle bonding. This finding is consistent with the aforementioned results derived from PI and CEC values.

### 2.11. Analysis of the Effect Mechanism of H_3_PO_4_ on Bentonite Properties

**Figure 8:**
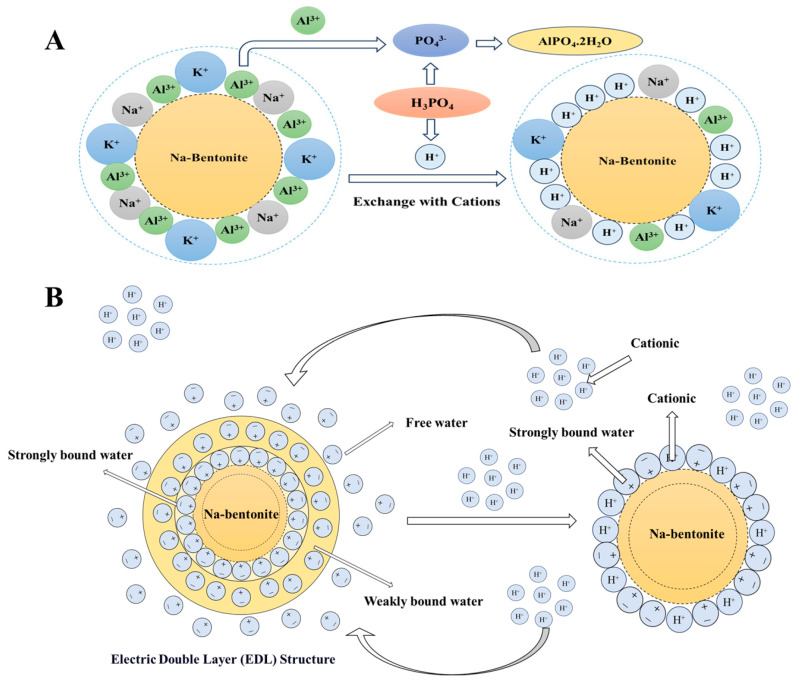
The mechanistic diagram of the interaction of H_3_PO_4_ with bentonite (**A**) Schematic Diagram of Ion Exchange and Adsorption (**B**) Electric Double Layer change schematic.

In summary, H_3_PO_4_ plays a pivotal role in enhancing the strength of sodium bentonite, as illustrated in Figure 8. As shown in Figure 8A, H_3_PO_4_ has a pH value of approximately 2, indicating its strong acidic properties. The hydrolyzed solution of H_3_PO_4_ contains a substantial amount of strongly acidic cations (primarily H_3_O^+^) and anions, with anions including species such as PO_4_^3−^. The primary interaction with sodium bentonite particles involves the high concentration of H_3_O^+^ cations. The surface of sodium bentonite particles is abundant in various high-value cations like aluminum ions (Al^3+^), sodium ions (Na^+^), and potassium ions (K^+^), along with polar water molecules and other substances. In an ion-exchange reaction, a significant number of H_3_O⁺ cations adsorb onto the sodium bentonite particles and replace the high-value cations and polar water molecules in the adsorption layer. This results in a decrease in potential and a thinning of the double electric layer. As depicted in Figure 8B, the ionization of H_3_PO_4_ produces strong cations, H_3_O^+^, which, under the influence of electrostatic forces, accumulate around the interface of the sodium bentonite particles. The displacement of polar water molecules from the surface of sodium bentonite occupies the effective potential sites on the particle surface, reducing the probability of polar water molecules and other cations binding to the particles. Consequently, the thickness of the double electric layer on the surface of the sodium bentonite particles decreases. This process not only significantly reduces the expansiveness of the bentonite layers but also facilitates the transformation of strongly bound water into weakly bound or free water on the surface of sodium bentonite. Therefore, under mechanical pressure, water can be expelled from within the clay, causing the bentonite particles to compact more closely together.

## 3. Materials and Methods

### 3.1. Materials

In this study, sodium bentonite was obtained from a factory located in Lingshou County, situated in the western region of Hebei province, with the chemical composition presented in Table 3. The fundamental properties of sodium bentonite were determined following the guidelines outlined in Standard for Geotechnical Testing Method (GB/T 50123-2019 [32]), as presented in Table 4. Based on the Unified Soil Classification System, sodium bentonite was classified as a high liquid limit soil (CH). H_3_PO_4_ was purchased from Sinopharm Chemical Reagent Co., Ltd. (Shanghai, China) and used in the experiment to stabilize the expansive soils.

### 3.2. Methods

In this study, the fundamental characteristics of various sodium bentonite, containing 1%, 2%, 3%, 4%, 6%, and 8% H_3_PO_4_, respectively, were determined using a limit water content test and compaction test. The effect of H_3_PO_4_ on the mechanical strength of sodium bentonite was investigated using the unconfined compression test (UCS). The electronic conductivity and pH of sodium bentonite were measured to indicate changes in the physical properties. Direct shear experiments were conducted to observe variations in the internal friction angle and cohesion of different sodium bentonites. X-ray diffraction (XRD), Fourier-transform infrared (FTIR), and scanning electron microscopy (SEM) analyses were performed to examine the material composition and microscopic structure of sodium bentonite with optimal mixing proportions.

#### 3.2.1. Sample Preparation

The compaction tests were conducted on sodium bentonite samples with H_3_PO_4_ in accordance with the Standard for Test Procedures of Soils for Highway Civil Engineering (JTG3430-2020 [33]), with results presented in Table 5. Depending on the requirements of pavement construction, the subgrade is typically compacted to a dry density unit that closely approximates the optimum condition [8]. Therefore, all samples in this study were prepared at their respective optimum moisture content and maximum dry unit weight values. Based on the desired optimum moisture content values, water or H_3_PO_4_ solution was thoroughly mixed with sodium bentonite, respectively. The mixture was then sealed in a polypropylene container using cling film and cured for 24 h to ensure uniform distribution of moisture within the sodium bentonite.

#### 3.2.2. Limit Water Content Test

During the test procedure, the sodium bentonite was sieved through a 0.5 mm standard sieve using a digital liquid-plastic limit combined tester manufactured by Cangzhou Xinke Construction Instrument Co., Ltd. (model LP-100D, Cangzhou, China). The standard sieve model was purchased from the Shaoxing Shangyu Shengchao Instrument Co., Ltd. (Shaoxing, China), and is in accordance with the national standard T6003.1-2022 [34]. During the experiment, the soil’s water content was measured in two stages: from a semi-solid state to a plastic state (plastic limit), and from a plastic state to a liquid state (liquid limit). A cone with dimensions of φ 50 × 50 mm and weighing 76 g was utilized. The plastic limit was defined as a cone depth of 2 mm, while the liquid limit was defined as a cone depth of 17 mm.

#### 3.2.3. Compaction Tests

In accordance with the standardized procedures outlined in the Test Procedures of Soils for Highway Civil Engineering (JTG 3430-2020 [33]), compaction tests were conducted using the Multifunctional Electric Compaction Tester manufactured by Beijing Zhongjiao Jianyi Technology Development Co., Ltd. (DZY-III, Beijing, China). Various dosages of H_3_PO_4_ were utilized in these tests to determine the maximum dry density and optimum moisture content. The size of the mold with the size of φ 100 × 127 mm was used, employing a 2.5 kg hammer that fell 27 times from a height of 300 mm. The weight of the samples was determined by weighing them after the compaction test.

#### 3.2.4. Physio-Chemical Tests

The pH and electrical conductivity (EC) tests were conducted to monitor the interaction between the additive and soil particles. For both tests, 20 g of dried sample was utilized after being passed through a sieve with a 40-mesh aperture. Subsequently, 100 mL of distilled water was introduced to the sodium bentonite. The mixture was vigorously shaken for 30 s at regular intervals, every 10 min. After 60 min, the pH and electrical conductivity values of the mixture were determined by using a pH meter Beijing Ouxin Sheng Technology CO., LTD. (PHS-3C, Shanghai, China) and an EC meter from Eutech Instruments (Eutech PC 2700, Waltham, MA, USA), respectively.

#### 3.2.5. Cation Exchange Capacity

The cation exchange capacity was determined by the methylene blue method. Then, 2 g of dry sample was placed in a beaker and mixed with 300 mL of water. The mixture was then stirred thoroughly to form a suspension. The pH was adjusted with sulfuric acid in the range of 2.5 to 3. Subsequently, a methylene blue solution was introduced into the beaker. A drop of mud was then extracted using either a burette or glass rod and carefully placed on the edge of the filter paper until the suspension in the beaker reached a titration point, resulting in a distinct blue spot surrounded by a light blue halo on the filter paper. The volume of methylene blue solution added was recorded for the subsequent calculation of cation exchange in the sample.

#### 3.2.6. Zeta Potential Test

A zeta potential test was conducted using a nanoparticle size analyzer from HORIBA, Ltd. (SZ-100, Kyoto, Japan), which was sonicated for 5 min and the supernatant was collected for analysis.

#### 3.2.7. Free Swelling Test

In accordance with the GB/T50123-2019 [32] geotechnical test method standard, 100 g of air-dried soil samples were taken through 0.5 mm sieve and oven baked at 105–110 °C until constant weight. A certain volume of dried soil sample was weighed using a free swell meter and added to a measuring cylinder containing 5 mL of 5% NaCl solution and 30 mL of pure water. Then, water was added into the maximum scale of the measuring cylinder and stirred. It was allowed to stand, and readings were taken at intervals of 2 h while ensuring that the difference in readings did not exceed 0.2 mL in 6 h.

#### 3.2.8. Unconfined Compression Test

The results of unconfined compressive strength experiments are typically utilized to evaluate the efficacy of a curing agent in soil stabilization. Consequently, the unconfined compressive strength data were employed in this study to assess the influence of varying H_3_PO_4_ dosages on the mechanical properties of the soil. In accordance with GB/T 50123-2019 Standard [32] for geotechnical testing methods, specimens were prepared under conditions that corresponded to their respective maximum dry density and optimum water content, with a specimen size of φ 50 × 100 mm. The specimens were weighed and measured after molding and subsequently cured for 7 days under standard curing conditions (temperature: 20 ± 2 °C, humidity: 95 ± 3%). The UCS experiment was conducted using a microcomputer-controlled automatic compressive and flexural machine from Jinan Hengsi Shengda Instrument Co., Ltd. (YAW-300D/10D, Jinan, China). The axial loading rate was set at 1 mm/min, and the experiment was terminated upon reaching a strain of 20%.

#### 3.2.9. Direct Shear Experiments

According to the Test Procedures of Soils for Highway Civil Engineering (JTG 3430-2020 [33]), direct shear experiments were conducted on φ 60 × 20 mm specimens under their respective optimal moisture content and maximum dry density conditions, by using a strain-controlled straight shear instrument from Road Instrument Branch of Nanjing Soil Instrument Factory Co., Ltd. (ZJ, Nanjing, China). The shear rate was set at 0.8 mm/min, and the load was applied at levels of 100, 200, 300, and 400 kPa. A total of 100 trials were conducted, and the experiment was concluded when no significant changes or oscillations were observed.

#### 3.2.10. FTIR and XRD

The treatment of sodium bentonite with H_3_PO_4_ led to the formation of novel compounds. These products can be characterized using X-ray diffraction (XRD) techniques [35,36]. XRD is a method employed for identifying unknown crystalline minerals [37]. Accurate mineral identification is achieved by comparing the sample’s diffraction peaks to a database of known substances. To determine the presence or absence of new minerals, sodium bentonite treated at optimum content of H_3_PO_4_ were compared with the blank sodium bentonite by observing changes in their diffraction peaks. The untreated and treated samples were analyzed using a X-ray diffractometer from Rigaku Corporation (Smartlab SE, Tokyo, Japan). The scanning angle ranged from 2θ = 5–60° with a step size of 0.01°. The incorporation of functional groups from H_3_PO_4_ was investigated using a Smart Fourier Transform Infrared Spectrometer from Thermo Fisher Scientific (Nicolet iS5, Waltham, MA, USA).

#### 3.2.11. SEM

Scanning electron microscopy (SEM) images of untreated and treated sodium bentonite were observed by a field emission scanning electron microscope from Carl Zeiss AG (Gemini 300, Oberkochen, BW, Germany) and used to assess the binding between sodium bentonite and H_3_PO_4_. A rectangular specimen with the size of 10 mm ×10 mm ×5 mm was extracted from the center of the specimen following the UCS test. Subsequently, it underwent a freeze-drying process for 12 h and was delicately broken using tweezers to obtain an intact and undisturbed structural surface, with the structural surface positioned vertically upwards. The specimen was then affixed onto a small copper table using conductive adhesive and coated with gold on its surface to enhance its electrical conductivity. Digital photographs were captured at various magnifications to observe microscopic morphology.

## 4. Conclusions

In this study, the addition of H_3_PO_4_ markedly enhanced the mechanical strength of sodium bentonite while effectively mitigating its expansivity. Specifically, the choice of 2% H_3_PO_4_ concentration was based on its optimal performance across all evaluated properties. At this concentration, the most significant improvement in the main engineering properties of the modified bentonite was observed: the plasticity index was the smallest, with a reduction of 39.9%; the UCS and shear strength reached their maximum values of 0.447 MPa and 95.38 MPa, respectively, which were 92.24% and 165% higher compared to the blanks; and the free swell rate was 80%, which was 23.8% lower than the blanks. The zeta potential indicated a substantial reduction in the double electric layer thickness. These results suggest that 2% H_3_PO_4_ provides an effective balance between performance enhancement and practical applicability. Changes in pH and conductivity confirmed substantial ion exchange between H_3_PO_4_ and sodium bentonite, promoting closer particle proximity. The marked reduction in PI and zeta potential indicated that H_3_PO_4_ decreased the thickness of the double electric layer, leading to tighter binding between clay particles. The formation of water-insoluble aluminum phosphate, as confirmed by XRD patterns, further contributed to improved soil compactness. The decrease in the total cation exchange capacity (CEC) provided direct evidence of ion exchange between H_3_PO_4_ and sodium bentonite, resulting in reduced surface hydration energy and denser soil particles. This manifested as decreased hydrophilicity in the water–clay system, leading to minimal free expansion volume of the particles. These findings demonstrate that H_3_PO_4_ as a curing agent has a significant positive impact on the expansivity and strength of sodium bentonite. Therefore, this research offers valuable insights for the development and application of environmentally friendly, cost-effective, and efficient expansive soil stabilizers.

## Figures and Tables

**Table 1:** Physical properties of bentonite containing H_3_PO_4_ with different dosages.

H_3_PO_4_ Dosage (%)	LL (%)	PL (%)	PI (%)
1.0	84.4	29.6	54.8
2.0	82.4	29.5	52.9
3.0	83.4	29.6	53.8
4.0	83.9	29.7	54.2
6.0	83.4	28.5	54.9
8.0	85.1	29.0	56.1

**Table 2:** Free swelling rate of sodium bentonite containing H_3_PO_4_ with different dosages.

H_3_PO_4_ Dosage (%)	Free Swelling Rate (%)
1.0	90
2.0	80
3.0	78
4.0	75
6.0	75
8.0	75

**Table 3:** Chemical composition of sodium bentonite.

Constituent	Percentage Present/%
SiO_2_	71.28
Al_2_O_3_	23.8
Fe_2_O_3_	1.68
Na_2_O	1.98
MgO	1.50
TiO_2_	0.08
CaO	1.20
K_2_O	0.42

**Table 4:** Basic engineering properties of sodium bentonite.

Property	Values
Liquid limit (%)	112.2
Plastic limit (%)	24.2
Plasticity index (%)	88.0
Free swelling rate (%)	105
pH	9.33
Optimum water content (%)	26.0
Maximum dry density (g/cm^3^)	1.47
Sand% (>0.075 mm)	10.89
Silt% (0.005–0.075 mm)	40.50
Soil% (<0.005 mm)	48.61
Soil type	CH

**Table 5:** Physical properties of sodium bentonite containing H_3_PO_4_ with different dosages.

H_3_PO_4_ Dosage (%)	w_opt_ (%)	MDD (g/cm^3^)
1.0	24.9	1.497
2.0	23.2	1.530
3.0	23.5	1.480
4.0	24.9	1.463
6.0	26.0	1.439
8.0	29.0	1.415

## Data Availability

The data presented in this study are available in the article.
